# Poems by Kumar

**DOI:** 10.4103/0019-5545.42407

**Published:** 2008

**Authors:** Vinay Kumar

**Affiliations:** Consultant Psychiatrist, Manoved Mind Hospital & Research Center Pvt. Ltd. NC-116, S.B.I. Officers Colony, Kankarbagh, Patna – 20, India. E-mail: dr_vinayk@yahoo.co.in; Translated from Hindi by: Dr. Pramod Kr. Singh, Professor & Head Dept. of Psychiatry, Patna Medical College, Patna, India

## THE NON-METALLIC AGE

Somnolence has spread and spilled all around,
Day has shrunk to patches of dusky islands,
Instead of returning unsolicited
After unwelcome knocks on the closed windows,
Sun now enters her room unhindered, unquestioned.
But Sunetra's eyes
Remain ever-closed,
Like the heavy shutters of a mega-shop.
The sun-rays keep beholding her
For long and long after,
Until they return defeated, unrewarded.

The somnolence fluid is spreading again, all-round
Astride the first day-break island.
Sunetra ruminates,
What days are these?
So dark and dusky,
All faces are blurred.
Fair ones have all blackened,
All voices have blunted tones.
Even ghostess-kids have
Now hoarse and husky voice.

My sensibility has strangely
Become pachydermic.
Is it a shield or a skin-cast?
Mom, tell me
When shall I come out of it?
Ask your doctor,
Promptly and dutifully
Replies my mother.
Doctor is even more diplomatic.
Says, a little while more.

Impatiently one day Sunetra shrieks.
I can no more live like this.
I want to first-hand-experience
The sheen-n-shine of all objects around.
I want to feel and hear
The down-pouring sun-rays
From the skies.
I want to dance to the tunes
Of metallic sounds.

O'Lord,
When shall it cease,
The dampened and dusky
Non-metallic age.

And one day
She stops all medicines.

## SUNETRA'S MOTHER

It is tough to be a mother.
Tougher still is to mother a child.
But toughest truly is
To keep mothering a Sunetra.

The flawless flow of life,
Afloat for
Twenty years and nine months.
Can it really end so quietly?
Insidiously, can
What time wrote for years,
Be proven wrong
In a whimper?
I am lost,
Totally lost.
My confidence is shattered.
I lose faith,
Both in pen and prose.

Transition of a mother
Is terribly tedious,
Into Sunetra's mother.
No less than the surreptitious
Sneaking-in of Schizophrenia.
A fifty-year-old woman
Becomes a dwelling
With walls made of pooled-up courage,
Windows closed to
All contacts and coteries,
Doors disinclined to welcome
One and all,
Inside she stands
Immersed up to chin
In pools of apprehension.
Any slight movement
Further threatens her nose.
After all
Her Mind,
Which essentially she is,
Cannot start swimming
Like a fish
In a flash.

Mothering Sunetra
Means
A drowning mother,
Trying to save
Her drowning daughter,
By over-inflating her lungs,
Completely oblivious of
Any possible rupture.

Mothering Sunetra
Also means
Losing oneself completely,
In the expedition
Of trying to save
A 'meaning'
That is just on the verge of
Fading away,
And living like
Siamese-twins
With your daughter.

Sunetra's world
Hovers inside the courtyard
Like a helicopter,
With mother hanging
All alone
By the lone hope
That surely her daughter
Shall break out one day
Of this crooked clamshell,
And run
And crawl on her knees,
Sporting and smearing dust-n-sand
Amidst a bunch of butterflies.

Sunetra's mother
Personifies
The ceaseless, tiresome, hard labor,
To enable her to buy
A 'palanquin' made of
Light and color and melody
For her daughter.

[ED.: *Sunetra is a semi fictional case of Paranoid Schizophrenia and the protagonist of an ongoing series of poems still in the process of being completed. A sample of two poems is being presented here.*]

